# Obtaining Spheroplasts of Armored Dinoflagellates and First Single-Channel Recordings of Their Ion Channels Using Patch-Clamping

**DOI:** 10.3390/md12094743

**Published:** 2014-09-05

**Authors:** Ilya Pozdnyakov, Olga Matantseva, Yuri Negulyaev, Sergei Skarlato

**Affiliations:** 1 Institute of Cytology, Russian Academy of Sciences, Tikhoretsky Ave. 4, St. Petersburg 194064, Russia; E-Mails: matantseva@cytspb.rssi.ru (O.M.); yurineg@mail.cytspb.rssi.ru (Y.N.); s_skarlato@yahoo.com (S.S.); 2 Department of Medical Physics, St. Petersburg State Polytechnical University, Polytechnicheskaya ul. 29, St. Petersburg 195251, Russia

**Keywords:** dinoflagellates, ion channels, patch-clamp, spheroplasts

## Abstract

Ion channels are tightly involved in various aspects of cell physiology, including cell signaling, proliferation, motility, endo- and exo-cytosis. They may be involved in toxin production and release by marine dinoflagellates, as well as harmful algal bloom proliferation. So far, the patch-clamp technique, which is the most powerful method to study the activity of ion channels, has not been applied to dinoflagellate cells, due to their complex cellulose-containing cell coverings. In this paper, we describe a new approach to overcome this problem, based on the preparation of spheroplasts from armored bloom-forming dinoflagellate *Prorocentrum minimum*. We treated the cells of *P. minimum* with a cellulose synthesis inhibitor, 2,6-dichlorobenzonitrile (DCB), and found out that it could also induce ecdysis and arrest cell shape maintenance in these microalgae. Treatment with 100–250 µM DCB led to an acceptable 10% yield of *P. minimum* spheroplasts and was independent of the incubation time in the range of 1–5 days. We show that such spheroplasts are suitable for patch-clamping in the cell-attached mode and can form 1–10 GOhm patch contact with a glass micropipette, allowing recording of ion channel activity. The first single-channel recordings of dinoflagellate ion channels are presented.

## 1. Introduction

Ion channels are protein complexes, which control the flow of ions down their electrochemical gradient through the cell membranes. These membrane proteins are crucial for all basic cellular processes, ranging from cell signaling to motility. Ion channels of animals have been extensively studied for many decades, and a vast array of information about their diversity, structure and functioning has been accumulated to date [1]. In contrast, ion channels of unicellular eukaryotes are considerably less studied, although they are not less important for protistan physiology [2]. For example, it has been shown that Ca^2+^ and Ca^2+^-dependent K^+^ and Na^+^ channels control the motile behavior of paramecia and *Chlamydomonas* sp. [3,4,5], and H^+^ channels are responsible for activation of bioluminescence in dinoflagellates [6,7] and involved in pH homeostasis of coccolithophores [8].

Dinoflagellates are ecologically important organisms with unique physiological and biochemical features [9]. Investigation of their ion channels represents one of the most attractive research areas, because these membrane proteins play an essential role in cell processes, such as cell signaling, proliferation and apoptosis, endo- and exocytosis, *etc*. [1,10,11]. Hence, ion channels may be involved in toxin production and release by marine dinoflagellates, as well as in harmful algal bloom proliferation. In this research, we focused on the potentially toxic bloom-forming dinoflagellate species, *Prorocentrum minimum*, a model object for studying the ecology and physiology of marine phytoplankton.

**Figure 1:**
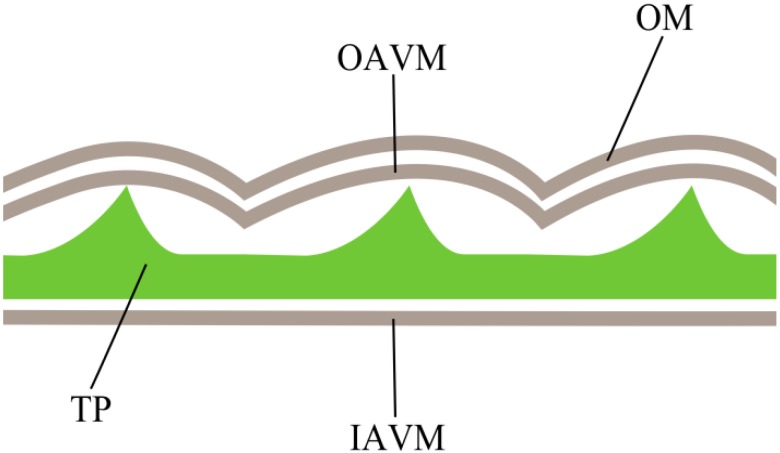
The structure of amphiesm a in *P. minimum*. OM, outermost membrane (plasma membrane); OAVM, outer amphiesmal vesicle membrane; TP, thecal plates; IAVM, inner amphiesmal vesicle membrane. Adapted from [17].

The patch-clamp technique is a powerful method for ion channel investigation. The remarkable advantage of this approach is an ability to investigate biophysical parameters of channel activity at a single-channel level. The patch-clamp technique in the cell-attached mode represents the formation of a tight contact between a recording micropipette and the cell membrane, called a patch, coupled to voltage clamping of this contact [12]. The accessibility of a membrane is an important prerequisite for successful patch formation; therefore, this electrophysiological method is easily applicable to many animal cells. In the case of the cell wall-containing plant and fungi cells, it is necessary to produce protoplasts or spheroplasts completely or partially lacking cell walls by means of enzymatic treatment [13,14]. However, this method would not work in the case of cellulose-containing dinoflagellates. Armored dinoflagellates possess a very complex cell covering, called an amphiesma. The amphiesma consists of four layers: outermost (plasma) membrane, outer amphiesmal vesicle membrane, inner amphiesmal vesicle membrane and cellulose thecal plates between them, which make cell covering very rigid [15,16]. Thecal plates of *Prorocentrum* species form spines sticking out of the cell surface [17] (Figure 1). On the one hand, this prevents tight contact formation, although the plasma membrane occurs outside a cell wall. On the other hand, hydrolytic enzymes cannot be used to digest cellulose, because thecal plates are protected by two layers of membranes.

We aimed at single-channel recordings of dinoflagellate ion channels and, therefore, searched for a method of obtaining the spheroplasts of dinoflagellates *P. minimum* amenable to patch-clamp studies. Dinoflagellates are able to discard the upper layers of their cell covering in response to stressing conditions. This process is called ecdysis; it can be triggered by a number of factors, including low osmolarity and temperature, nutrients depletion and exposure to certain chemical agents [18,19,20,21]. Ecdysed cells, widely known in the literature as pellicle cysts [22], represent spheroplasts. Furthermore, spheroplasts can be produced by impairing the process of thecal plate formation. Kwok *et al.* in 2007 obtained spheroplasts of a dinoflagellate *Crypthecodinium cohnii* using polyethylene glycol and observed a decreased amount of cellulose in the treated cells [23]. Nevertheless, their findings can hardly be effectively used in electrophysiological studies, because polyethylene glycol dramatically changes the properties of membranes, e.g., it induces their fusion, and, thus, may also affect ion channel activity.

In the present study, we tested two approaches to obtain the spheroplasts of dinoflagellates *P. minimum*, which would not have a direct effect on membranes. One involved the triggering of ecdysis by physical stress conditions (centrifugation), whereas the other employed chemical treatment with 2,6-dichlorobenzonitrile (DCB), a well-known inhibitor of cellulose synthesis [24].

## 2. Results and Discussion

### 2.1. Ecdysed Cells

**Figure 2:**
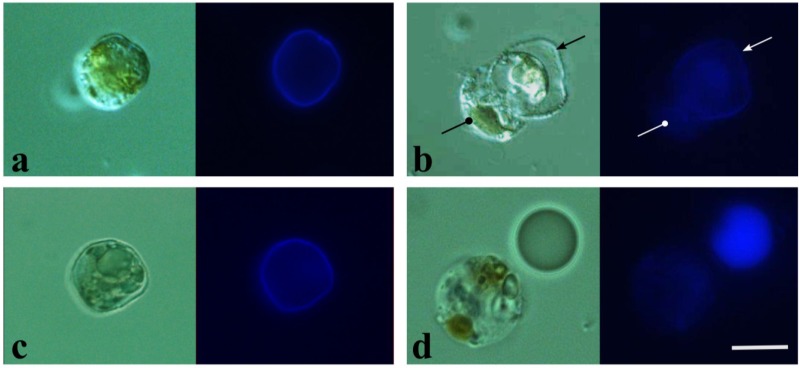
Photomicrographs of Calcofluor White M2R (CFW)-stained cells of *P. minimum*. (**Left image**) Phase contrast image; (**right image**) UV-light image visualizing cellulose. The scale bar represents 10 µm. (**a**) Untreated cell; (**b**) ecdysing cell (circle-headed arrow) discarding its theca (triangle-headed arrow); ecdysis was triggered by centrifugation (5 min at 10,000 rcf); (**c**) cell treated with DMSO; (**d**) 2,6-dichlorobenzonitrile (DCB)-induced spheroplast and fluorosphere used as a fluorescence standard.

When exposed to stressing factors, e.g., centrifugation, a dinoflagellate discards the upper layers of its cell covering, namely, the outermost plasma membrane, outer amphiesmal vesicle membrane and cellulose thecal plates. At the same time, the inner amphiesmal vesicle membrane turns into the new plasma membrane, which is not underlined by a thick and rigid cell wall [25]. Indeed, staining with Calcofluor White M2R (CFW) showed a sufficient decrease in the cellulose content of ecdysed cells as compared to untreated cells (Figure 2a,b). We used this phenomenon in order to patch-clamp just ecdysed *P. minimum* cells, taking into account that they must be more accessible for a micropipette. Nevertheless, we could not obtain any patches with such cells that seemed to be as rigid as untreated ones. When a micropipette touched such cells, no resistance response was obtained (resistance remained at the pipette resistance level of 8–15 MOhm).

### 2.2. DCB-Induced Spheroplasts

Treatment of a *P. minimum* culture with DCB (final concentration 50–300 µM) over 1–5 days led to the spheroplasts’ formation. Obtained spheroplasts were 7–20 µm in diameter, had a spherical shape and, thus, could be distinguished from the other, saucer-like cells. CWF staining showed relatively low cellulose content in DCB-induced spheroplasts, as compared to cells treated with 5–30 mM DMSO, a solvent used to make the DCB solution (Figure 2c,d). Such spherical cells appeared to be suitable for patch-clamping. Briefly, after a micropipette contacted a spheroplast, suction was applied until the seal resistance reached 500–600 MOhm. In most cases, after that point, patches were rapidly formed, with the resistance ranging from 1 to 10 GOhm. The achieved resistance values were sufficient for recording the ion channel activity at a single-channel level (cell-attached mode).

DCB-induced spheroplasts were able to restore motility, proving their viability, and an active physiological state. Our results support the previous observation that DCB is not toxic for dinoflagellates, but only delays a cell cycle at the G_1_ phase, which is coupled to cellulose synthesis in these organisms [26]. Thus, we believe that DCB can be readily used in electrophysiological studies without the danger of introducing artifacts.

**Figure 3:**
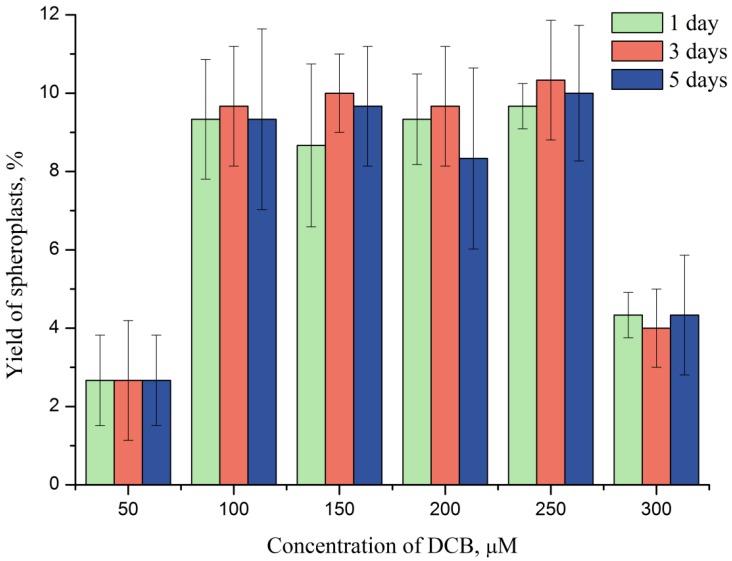
The yield of spheroplasts of *P. minimum* depending on DCB concentration and time of incubation.

The number of successful patches is always lower than the number of patch-clamping attempts. Hence, to increase the effectiveness of the method, the maximal spheroplasts harvest has to be achieved. We tested a range of DCB concentrations and incubation periods in order to find conditions providing the highest yield of spheroplasts. The maximal yield of spheroplasts was 9.5% ± 1.5% (mean ± SD) of the total cell number (Figure 3). This result was achieved at 100–250 µM DCB irrespectively of the incubation time in the tested range (1–5 days). Furthermore, the harvest of spheroplasts was independent of the culture growth state. Although the maximal yield of spheroplasts of about 10% does not seem very high, it is absolutely sufficient for patch-clamping. For instance, if the cell density of the stock culture is 4 × 10^4^ cells mL^−1^, one can expect the harvest of the potential patch-clamp target cells of 4 × 10^3^ cells mL^−1^, which is more than is possible to patch during one day.

### 2.3. DCB as an Inducer of Ecdysis in P. minimum

**Figure 4:**
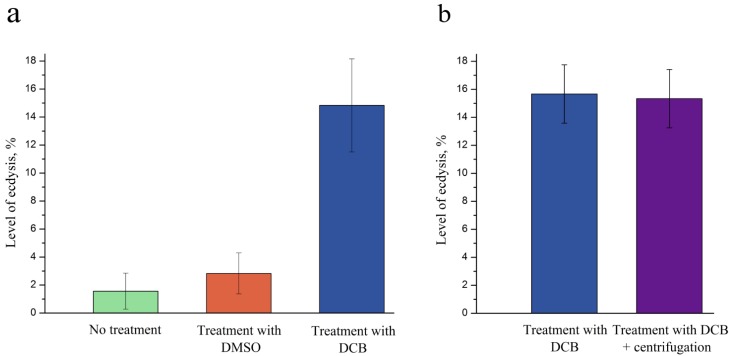
Level of ecdysis in the culture of the dinoflagellate, *P. minimum*, depending on the treatment (**a**) No treatment, treatment with 10 mM DMSO and treatment with 100 µM DCB; (**b**) Treatment with 100 µM DCB and treatment with 100 µM DCB combined with centrifugation*.*

We found that the level of ecdysis in *P. minimum* was much higher in DCB-treated cells than in untreated cells or cells treated with 10 mM DMSO (Figure 4a). Ecdysis induced by DCB started after 4 h following the addition of 100 µM DCB to a cell culture; its level achieved the maximum after 20 h, and from that time, stayed more or less invariable. Therefore, we suggest that DCB represents a stress agent, inducing ecdysis in *P. minimum* and possibly other dinoflagellates. Most probably, the process of the spheroplasts’ formation occurs as a result of both cellulose synthesis inhibition and discarding of the old cell walls by DCB-treated cells.

Since ecdysis in *P. minimum* can also be induced by centrifugation, we expected an increase in the yield of spheroplasts after additional centrifugation. The cells of *P. minimum* were treated with 100 µM DCB during 24 h and then centrifuged for 5 min at 10,000 rcf 2.5 h prior to counting. However, the yield of spheroplasts and the level of ecdysis were at the same levels as in the control without centrifugation (Figure 4b). We suppose that ecdysis itself is a necessary, though insufficient condition for the spheroplasts formation. Presumably only a certain fraction of a population is capable of ecdysis, and this process is independent of the number of triggering stress factors.

### 2.4. Ecdysed Cells vs. DCB-Induced Spheroplasts

**Figure 5:**
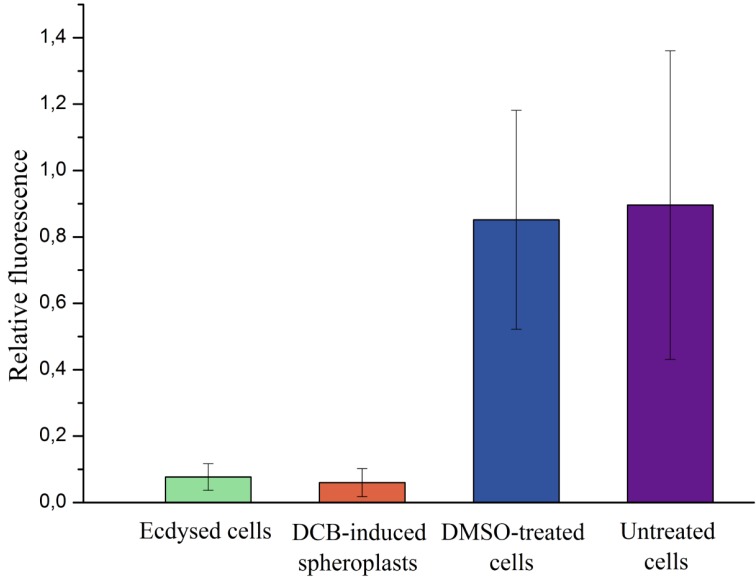
Relative fluorescence of CFW-stained ecdysed cells (*n* = 12), DCB-induced spheroplasts (100 µM DCB) (*n* = 17), DMSO-treated cells (10 mM) (*n* = 15) and untreated cells (*n* = 15) of the dinoflagellate, *P. minimum.*

Freshly-ecdysed cells obtained via centrifugation were not amenable to patch-clamping in contrast to DCB-induced spheroplasts. In order to explain this difference, we compared the amount of cellulose in both kinds of cells by means of CWF staining (Figure 2 and Figure 5). Surprisingly, the relative fluorescence of spheroplasts and ecdysed cells did not differ significantly (two-sample *t*-test: *p* > 0.2); hence, the cellulose contents of both types of cells were the same, and the rigid structure of ecdysed cells could not be explained by a thicker cellulose layer. Remarkably, the newly-ecdysed cells were flattened, similar to the untreated *P. minimum* cells, whereas DCB-induced spheroplasts were spherical. Apparently, a residual cellulose layer of DCB-induced spheroplasts could not maintain a native shape of a cell, suggesting that DCB not only prevented accumulation of cellulose, but also affected its arrangement. This assumption is supported by the literature data. DCB was shown to influence the structure and tensile strength of cell walls of higher plants in various ways [27]. Moreover, it caused disarrangement of the terminal complex systems responsible for the synthesis of cellulosic fibrils in the xanthophycean alga, *Vaucheria hamata* [28]. We assume that although ecdysed cells and DCB-induced spheroplasts possessed equal amounts of residual cellulose, its native organization might be disordered by DCB in the latter, making their cell coverings friable and more suitable for patch-clamping.

Our results suggest that the DCB-induced formation of spheroplasts occurs in the following sequence. Treatment with DCB induces cell covering shedding in *P. minimum*. At the same time, DCB inhibits synthesis of new cellulose, as well as cell shape maintenance. The resulting spherical cells are able to recover their motility; therefore, subsequent centrifugation is needed to get non-motile cells for patch-clamping.

### 2.5. First Single-Channel Recordings in Armored Dinoflagellates

**Figure 6:**
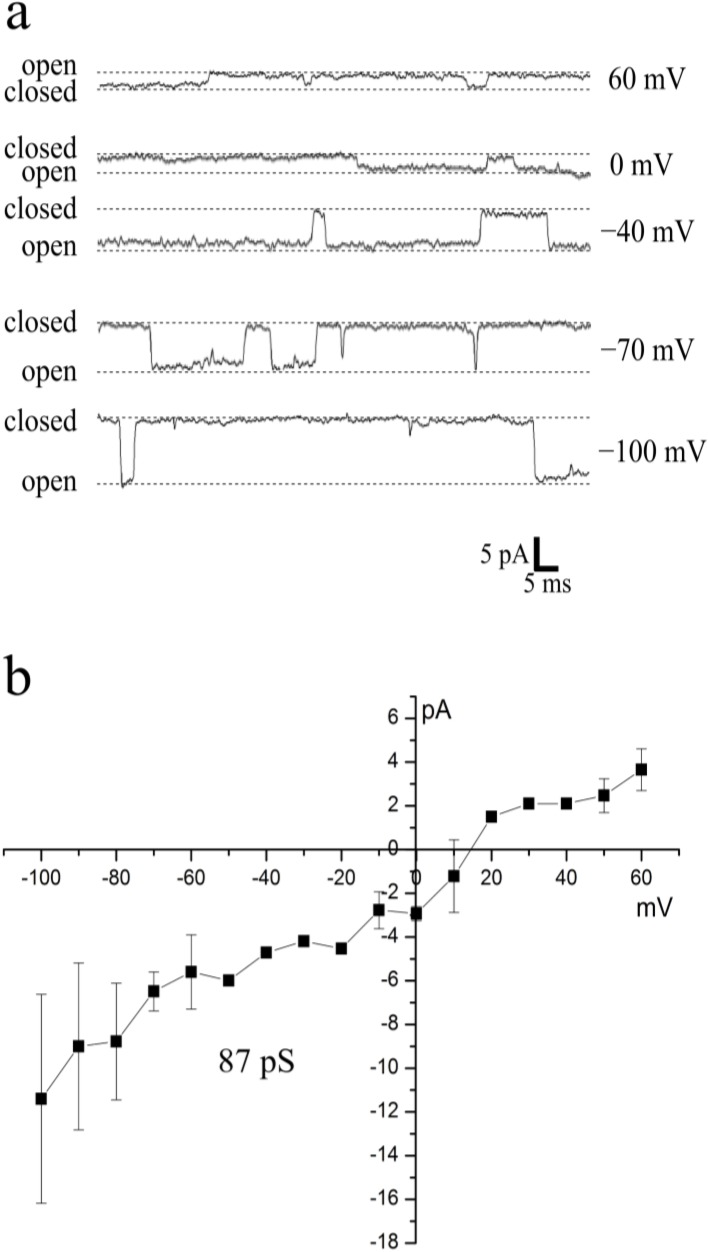
First single-channel recordings in the cell-attached mode and I-V curve of ion channels in the dinoflagellate *P. minimum.* The external solution contained 240 mM KCl, 2 mM CaCl_2_, 5 mM HEPES/Tris, pH 7.2. The pipette solution contained 240 mM NaCl, 2 mM CaCl_2_, 5 mM HEPES/Tris, pH 7.2. (**a**) Representative single-channel current traces at various voltages; currents were low-pass-filtered at 1 kHz and digitized at 5 kHz with an A/D converter; dotted lines indicate open and closed states of the channel; (**b**) Average current-voltage relationship estimating a conductance of 87 ± 4 pS (mean ± SD) and a reversal potential of +15 mV (*n* = 7).

We used DCB-induced spheroplasts for patch-clamping and, to our knowledge, received the first single-channel recordings of dinoflagellate ion channels (Figure 6a). During the process of ecdysis required for the spheroplasts formation, the inner amphiesmal vesicle membrane turns into the new plasma membrane [25]; therefore, we conclude that we patched the plasma membrane of *P. minimum*. However, it should be noted that there is no direct evidence that the plasma membrane of spheroplasts is identical to the plasma membrane of untreated vegetative cells.

Recordings were performed in the cell-attached mode at 240 mM NaCl in the pipette solution (240 mM NaCl, 2 mM CaCl_2_, 5 mM HEPES/Tris, pH 7.2) and 240 mM KCl in the external solution (240 mM KCl, 2 mM CaCl_2_, 5 mM HEPES/Tris, pH 7.2). The recorded ion channels had a rather high conductance of 87 ± 4 pS (mean ± SD, *n* = 7) and a reversal potential of +15 mV (Figure 6b). However, the absolute value of the membrane patch potential represents a sum of the command potential and the resting potential (E_rest_) that cannot be directly measured in the cell-attached mode [12]. Thus, the actual reversal potential could differ from the determined value if E_rest_ was nonzero during recording. Assuming that E_rest_ is set by K^+^ gradient and the internal K^+^ concentration of *P. minimum* is in the range of 120–240 mM, as it was reported for other species of marine microalgae [29,30], the actual value of the reversal potential could be +15 mV (if internal [K^+^] = 240 mM, E_rest_ = 0) or higher (if internal [K^+^] < 240 mM, E_rest_ > 0). At the present time, we cannot make reliable conclusions about the selectivity of the recorded channels. Further experiments will shed more light on this issue.

Voltage-sensitive Na^+^ and Cl^−^ channels were reported earlier in the dinoflagellate, *Noctiluca miliaris* [31,32]. The authors used the voltage-clamping technique, allowing investigations of the integral ion currents across cell membranes, but not the currents through the single channels. The first patch-clamp study of dinoflagellate ion channels (whole-cell mode) was performed by Smith *et al.* [7], who used a heterologous expression system (HEK-293 cells) in order to investigate voltage-gated H^+^ channels of *Karlodinium veneficum*. However, it is usually preferential to study proteins in the respective native organisms, since the unique physiology and molecular processing during gene expression specific to a heterologous system may uncontrollably affect their activity. Moreover, the gene coding voltage-gated H^+^ channels of *K. veneficum* identified by Smith *et al.* [7] is likely to be the only known sequence of an ion channel gene in dinoflagellates. The application of the latter technique for studies of other ion channels in these organisms will be possible only after identifying the respective genes and amino acid sequences. The method described in our paper has a great potential to facilitate profound ion channel studies of dinoflagellates and promote understanding of the ecological relevance and evolution of the structure and functions of ion channels in eukaryotes.

## 3. Experimental Section 

### 3.1. Culture Conditions

The culture of *Prorocentrum minimum* isolated from the Black Sea was obtained from the collection of Department of Hydrobiology, Lomonosov Moscow State University. The culture was grown at room temperature in 17 psu f/2 medium (half strength medium f) [33] without the addition of silicate on a 12 h light:12 h dark photoperiod under 50 µmol photons m^−2^ s^−1^.

### 3.2. Preparation of Spheroplasts

#### 3.2.1. Physical Treatment

Ecdysis of *P. minimum* was induced by centrifugation for 5 min at 10,000 rcf. After centrifugation, cells were resuspended in f/2 medium, incubated over 1.5–2 h and centrifuged again at the same conditions. A pellet was resuspended in the external recording solution 30 min before patch-clamping.

#### 3.2.2. Chemical Treatment

The 2,6-dichlorobenzonitrile (DCB) (Sigma-Aldrich, St. Louis, MO, USA) was dissolved in dimethyl sulfoxide (Sigma-Aldrich, St. Louis, MO, USA) and stored as a 10 mM stock solution at −20 °C. To induce the spheroplasts formation, 10 mM DCB solution was added to a *P. minimum* culture (10^4^–10^5^ cells mL^−1^) in 1.5 mL transparent centrifuge tubes to a final concentration 50–300 µM and gently mixed with it. Tubes containing treated cells were incubated on a 12 h light:12 h dark photoperiod over 1–5 days. Before the patch-clamp experiments, the tubes were centrifuged for 5 min at 10,000 rcf in order to make cells immotile and remove f/2 medium. A pellet was resuspended in the external recording solution 30 min before patch-clamping.

### 3.3. Patch-Clamp Procedures 

From 100 to 150 µL of cell-containing solution were placed undisturbed for two minutes into a chamber to allow cells to settle to the bottom of the chamber. External recording solution contained 240 mM KCl, 2 mM CaCl_2_, 5 mM HEPES/Tris, pH 7.2. Spheroplasts appeared as spherical cells with a diameter of 7–20 µm, lacking a distinctly visible cell wall. In the case of ecdysed cells, we tried to patch those that were still connected to their old thecae, since freshly-ecdysed cells were otherwise indistinguishable from the rest of the cells. We used borosilicate glass BF150-110-10 or BF150-86-10 (Sutter Instrument Company, Novato, CA, USA) to manufacture micropipettes with a resistance of 8–15 MOhm when filled with the pipette recording solution (240 mM NaCl, 2 mM CaCl_2_, 5 mM HEPES/Tris, pH 7.2). Membrane currents were measured using an Axopatch 200B patch-clamp amplifier (Axon Instruments/Molecular Devices, Eugene, OR, USA), low-pass-filtered at 1–2 kHz and digitized at 5 kHz with an A/D converter. Data were collected and analyzed with pClamp 9.2 (Axon Instruments/Molecular Devices, Eugene, OR, USA) and Origin 8.0 software (OriginLab Corp., Northampton, MA, USA). The experiments were performed in the cell-attached mode. To record membrane currents, we used a pulse protocol, which started at −70 mV (the holding potential) and stepped to the values from −100 mV to 60 mV in increments of 10 mV. The voltage sweeps had a duration of 100 ms. Recordings were performed at room temperature (22–25 °C).

### 3.4. Cell Counting and Calculation of the Spheroplasts Yield and Ecdysis Level

Cells and spheroplasts were counted in a Fuchs-Rosenthal counting chamber or using ocular grids with the known dimensions. The yield of spheroplasts was calculated as a ratio of spheroplasts to the total cell number expressed as a percentage. The level of ecdysis was calculated as a ratio of empty thecae to the total cell number expressed as a percentage.

### 3.5. Cellulose Staining and Microscopy

To assess the cellulose content of *P. minimum* cells and spheroplasts, they were stained with Calcofluor White M2R (Sigma-Aldrich, St. Louis, MO, USA), a fluorescent dye specific for cellulose and other β-linked polymers. A 1:1000 w/v stock solution made with PBS was directly added to a cell suspension at the ratio 1:15.

Stained cells were observed and photographed using the Axio Observer.Z1 (Carl Zeiss MicroImaging GmbH, Göttingen, Germany) inverted microscope with UV-illumination and a 565-nm excitation and a 445–450-nm emission filter at 100× magnification.

To normalize the fluorescence of different samples, the 10-µm diameter Flow-Check™ fluorospheres (Beckman Coulter, Pasadena, CA, USA) were used as a standard. The fluorospheres were directly added into a cell suspension before microscopic observations.

### 3.6. Image Analysis

Obtained images were analyzed with ImageJ software [34]. The fluorescence of *P. minimum* cells and fluorospheres was estimated as follows:

F = ID − (A × MFB)
(1)

where F is the fluorescence of a cell or fluorosphere, ID is the integrated density, A is the area of the selected cell or fluorosphere and MFB is the mean fluorescence of background readings. The relative fluorescence of DCB-induced spheroplasts and ecdysing cells was calculated as a ratio of the fluorescence of spheroplasts or ecdysing cells to the fluorescence of fluorospheres.

## 4. Conclusions 

Our work represents a successful step towards the investigation of dinoflagellate ion channels at the single-channel level. The application of the patch-clamp technique to armored dinoflagellates became possible owing to the effects of DCB on *P. minimum*. DCB not only inhibits cellulose biosynthesis, but also induces ecdysis and hampers the mature cell shape maintenance in this organism. We used DCB-induced spheroplasts and obtained the first single-channel recordings of ion channels in dinoflagellates. The described approach opens promising and so far unattainable possibilities for electrophysiological studies of these remarkable and environmentally important organisms.

## References

[1] Hille B. (2001). Ion Channels of Excitable Membranes.

[2] Martinac B., Saimi Y., Kung C. (2008). Ion channels in microbes. Physiol. Rev..

[3] Beck C., Uhl R. (1994). On the localization of voltage-sensitive calcium channels in the flagella of *Chlamydomonas reinhardtii*. J. Cell Biol..

[4] Saimi Y., Hinrichsen R.D., Forte M., Kung C. (1983). Mutant analysis shows that Ca^2+^-induced K^+^ current shuts off one type of excitation in *Paramecium*. Proc. Natl. Acad. Sci. USA.

[5] Kink J.A., Maley M.E., Preston R.R., Ling K.Y., Wallen-Friedman M.A., Saimi Y, Kung C. (1990). Mutation in paramecium calmodulin indicate functional differences between the *C*-terminal and *N*-terminal lobes *in vivo*. Cell.

[6] Eckert R., Sibaoka T. (1968). The flash-triggering action potential of luminescent dinoflagellate *Noctiluca*. J. Gen. Physiol..

[7] Smith S., Morgan D., Musset B., Cherny V., Place A., Woodland Hastings J., DeCoursey T. (2011). Voltage-gated proton channel in a dinoflagellate. Proc. Natl. Acad. Sci. USA.

[8] Taylor A.R., Chrachri A., Wheeler G., Goddard H., Brownlee C. (2011). A voltage-gated H^+^ channel underlying pH homeostasis in calcifying coccolithophores. PLoS Biol..

[9] Hackett J., Anderson D., Erdner D., Bhattacharya D. (2004). Dinoflagellates: A remarkable evolutionary experiment. Am. J. Bot..

[10] Urrego D., Tomczak A.P., Zahed F., Stühmer W., Pardo L.A. (2014). Potassium channels in cell cycle and cell proliferation. Phil. Trans. R. Soc. B.

[11] Bortner C.D., Cidlowsky J.A. (2014). Ion channels and apoptosis in cancer. Phil. Trans. R. Soc. B.

[12] Molleman A. (2003). Patch Clamping: An Introductory Guide to Patch Clamp Electrophysiology.

[13] Elzenga J.T.M., Keller C.P., van Volkenburgh E. (1991). Patch clamping protoplasts from vascular plants. Plant Physiol..

[14] Garrill A., Davies J.M. (1994). Patch clamping fungal membranes: A new perspective on ion transport. Mycol. Res..

[15] Morrill L.C., Loeblich A.R. (1983). Ultrastructure of the dinoflagellate amphiesma. Int. Rev. Cytol..

[16] Pozdnyakov I., Skarlato S. (2012). Dinoflagellate amphiesma at different stages of the life cycle. Protistology.

[17] Dodge J.D. (1965). Thecal fine-structure in the dinoflagellate genera *Prorocentrum* and *Excuvialle*. J. Mar. Biol. Ass. UK.

[18] Adamich M., Sweeney B.M. (1976). The preparation and characterization of *Gonyaulax* spheroplasts. Planta.

[19] Anderson D.M., Kulis D.M., Binder B.J. (1984). Sexuality and cyst formation in the dinoflagellate *Gonyaulax tamarensis*: Cyst yield in batch cultures. J. Phycol..

[20] Hardeland R. (1994). Induction of cyst formation by low temperature in the dinoflagellate *Gonyaulax polyedra* Stein: Dependence on circadian phase and requirement of light. Experientia.

[21] Wong J.T.Y., Wong Y.H. (1994). Indoleamine-induced encystment in dinoflagellates. J. Mar. Biol. Ass. UK.

[22] Bravo I., Figueroa R.I. (2014). Towards an ecological understanding of dinoflagellate cyst functions. Microorganisms.

[23] Kwok A.C.M., Mak C.C.M., Wong F.T.W., Wong J.T.Y. (2007). Novel method for preparing spheroplasts from cells with an internal cellulosic cell wall. Eukaryot. Cell.

[24] Mayer Y., Herth W. (1978). Chemical inhibition of cell wall formation and cytokinesis, but not of nuclear division, in protoplasts of *Nicotinia tabacum* L. cultured *in vitro*. Planta.

[25] Morrill L.C. (1984). Ecdysis and the location of the plasma membrane in the dinoflagellate *Heterocapsa niei*. Protoplasma.

[26] Kwok A.C.M., Wong J.T.Y. (2003). Cellulose synthesis is coupled to cell cycle progression at G_1_ in the dinoflagellate *Crypthecodinium cohnii*. Plant Physiol..

[27] Sheldletzky E., Shmuel M., Trainin T., Kalman S., Delmer D. (1992). Cell wall structure in cells adapted to growth on the cellulose-synthesis inhibitor 2,6-dichlorobenzonitrile. Plant Physiol..

[28] Mizuta S., Brown R.M. (1992). Effects of 2,6-dichlorobenzonitrile and tinopal LPW on the structure of the cellulose synthesizing complexes of *Vaucheria hamata*. Protoplasma.

[29] Dickson D.M., Kirst G.O. (1987). Osmotic adjustment in marine eukaryotic algae: The role of inorganic ions, quaternary ammonium, tertiary sulphonium and carbohydrate solutes. I. Diatoms and rhodophyte. New Phytol..

[30] Dickson D.M., Kirst G.O. (1987). Osmotic adjustment in marine eukaryotic algae: The role of inorganic ions, quaternary ammonium, tertiary sulphonium and carbohydrate solutes. II. Prasinophytes and haptophytes. New Phytol..

[31] Oami K., Sibaoka T., Naitoh Y. (1988). Tentacle regulating potentials in *Noctiluca miliaris*: Their generation sites and ionic mechanisms. J. Comp. Physiol. A.

[32] Oami K., Naitoh Y. (1990). Distribution of ion channels in the membrane of the dinoflagellate *Noctiluca miliaris*. J. Exp. Biol..

[33] Guillard R.R.L., Ryther J.H. (1962). Studies of marine diatoms I. *Cyclistella nana* Hustedt and *Detonula confervacea* (Cleve) Gran. Can. J. Microbiol..

[34] Rasband W.S. (2013). ImageJ, 1.47v.

